# Exploring the Potential of Lidocaine in a Bilateral Erector Spinae Plane Block for Multimodal Analgesia in Partial Hepatectomy: A Case Report

**DOI:** 10.7759/cureus.40047

**Published:** 2023-06-06

**Authors:** Heitor Medeiros, Sara Amaral, Raul Amorim, Wallace A Da Silva

**Affiliations:** 1 Department of Anaesthesiology, Hospital Universitario Onofre Lopes, Natal, BRA; 2 Department of Anaesthesiology, Hospital Regional Deputado Afonso Guizzo, Ararangua, BRA; 3 Department of Anaesthesiology, Hospital Universitário Onofre Lopes, Natal, BRA

**Keywords:** resource limitations, multimodal analgesia, lidocaine, erector spinae plane block, partial hepatectomy

## Abstract

The bilateral erector spinae plane block (ESP) has been effectively used for abdominal surgery, and the placement of catheters is known to extend the benefits of the block while allowing for the adjustment of local anesthetic doses as necessary. Since fascial plane blocks require high volumes of local anesthetic and a prolonged duration of effect, typically, long-acting local anesthetics are preferred. However, lidocaine is not commonly chosen for these types of blocks due to the large volumes required and the associated risk of local anesthetic systemic toxicity. Nonetheless, we present a case report of a patient who underwent a partial hepatectomy under general anesthesia, with perioperative placement of a bilateral ESP block. Bilateral catheters were inserted, and 1% lidocaine was selected as the local anesthetic due to resource limitations. The surgery proceeded without complications, and the patient reported effective analgesia and a high level of satisfaction. Our report suggests that the utilization of lidocaine in a continuous ESP block can be a successful alternative for partial hepatectomies.

## Introduction

Liver surgery is a procedure frequently accompanied by substantial postoperative pain, a high risk of bleeding, and potential coagulation disorders. As a result, there is increasing utilization of regional anesthesia techniques to achieve effective analgesia, reduce opioid consumption, and enhance hemodynamic stability compared to neuraxial techniques. Moreover, certain regional anesthesia procedures are performed peripherally and superficially, thereby avoiding contraindications associated with coagulation disorders that may arise in liver surgery.

Several regional anesthesia techniques are available, and one notable option is the erector spinae plane (ESP) block. Initially described by Forero et al. in 2016 [1], the ESP block has gained widespread popularity attributed to its straightforward approach, safety, and effectiveness.

In this case report, we present a patient who underwent a partial hepatectomy and received a bilateral ESP block with catheter placement as part of a multimodal analgesic approach for postoperative pain control. Given resource constraints, a 1% lidocaine solution was utilized for the ESP block. We outline the patient's clinical course, describe the ESP block technique employed, and evaluate its effectiveness in delivering analgesia.

The anesthetic and surgical procedures were executed without complications. The surgery lasted 10 hours, with catheters removed at the conclusion of the procedure by request from the surgical team. The patient was extubated four hours later in the intensive care unit.

## Case presentation

We present the case of a 53-year-old male patient, American Society of Anesthesiologists (ASA) physical status III, height 190 cm, 120 kg, and BMI 33.8, with a medical history of chronic hypertension and previous rectosigmoid adenocarcinoma. He underwent a rectosigmoidectomy two years prior, followed by a liver metastasectomy (segments V and IVb) approximately one year later. The patient was scheduled for a partial hepatectomy (segment VII), with Bleck and Portovac drain placement.

**Figure 1:**
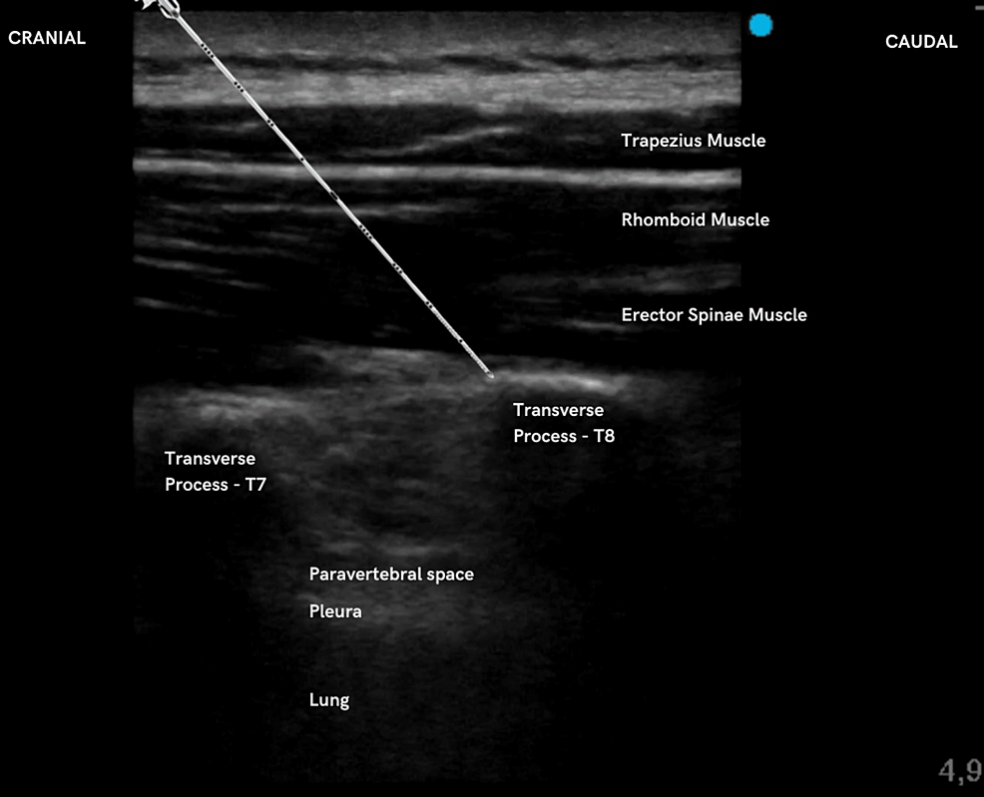
Ultrasound landmarks

Following the application of standard monitoring devices, the patient underwent a combined approach of general and regional anesthesia. Induction was done with a dosage of 1.7 mg/kg of propofol, 2.8 mcg/kg of fentanyl, and 0.2 mg/kg of cisatracurium. Maintenance was carried out with 1.8% sevoflurane at end-tidal concentration. Prior to the surgical incision, an ultrasound-guided bilateral ESP block was executed at the T8 level, with the patient positioned in the right lateral decubitus. A high frequency (6-12 MHz) linear probe, HD11XE ultrasound (Philips, Amsterdam, The Netherlands), was utilized, with planned visualization, adhering to a standard aseptic technique. Following the visualization of the transverse process, an 18-gauge Tuohy needle was inserted from the cephalad to the caudad direction, employing an in-plane approach. After making contact with the transverse process, the needle was slightly retracted, and 30 mL of 1% lidocaine was slowly administered to create a gap between the transverse process and the erector spinae muscle plane (Figure 1).

Subsequently, a catheter (Portex epidural catheter) was inserted 5 cm beyond the needle's tip at the same level and then secured at the skin. The procedure was replicated on the opposite side using identical positioning and technique. A 10 mL bolus of 1% lidocaine was subsequently administered every two hours on each side until the maximum dosage of 7.0 mg/kg was reached.

A multimodal analgesia regimen was implemented, which included a single dose of 0.2 mg/kg intravenous (IV) bolus of ketamine, a 1 mcg/kg IV bolus of clonidine, a 2 g IV bolus of metamizole, a 40 mg IV bolus of tenoxicam, and a 30 mg/kg IV bolus of magnesium sulfate, followed by an infusion of 10 mg/kg/h for two hours. Prophylaxis for postoperative nausea and vomiting (PONV) was done with an 8 mg IV bolus of ondansetron and a 4 mg IV bolus of dexamethasone.

The anesthetic and surgical procedures were executed without complications. The surgery lasted 10 hours, with catheters removed at the conclusion of the procedure by request from the surgical team. The patient was extubated four hours later in the intensive care unit.

**Figure 2:**
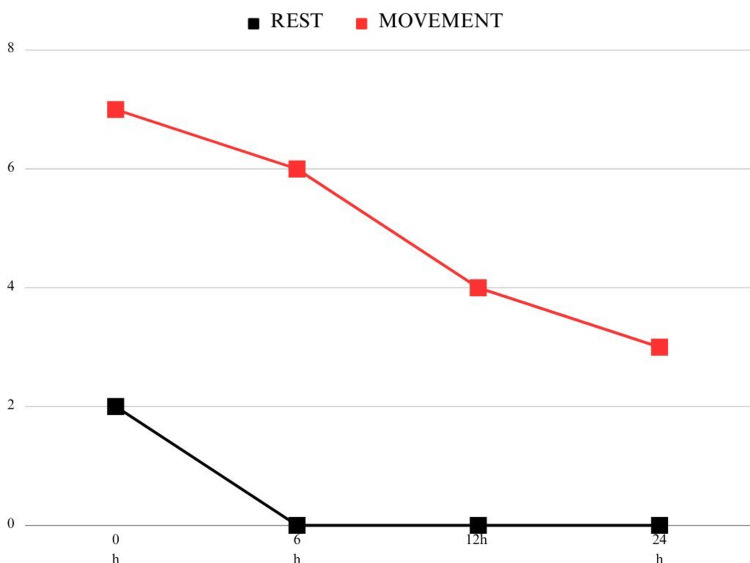
Pain scores using the visual analog scale

Pain scores were evaluated using the visual analog scale (VAS) immediately post-extubation and at 6, 12, and 24 hours postoperatively, both at rest and during movement. The pain scores at rest were low, ranging from 2, 0, 0, and 0 out of 10. In contrast, the scores registered during movement were 7, 6, 4, and 3 out of 10 within the same time intervals (Figure 2).

Consumption of opioids in the first 24 hours was documented. The patient used 10 mg of nalbuphine every eight hours in addition to fixed dipyrone every six hours postoperatively. He did not report any instances of PONV and expressed a high level of satisfaction with his analgesia following this operation as compared to his prior procedure. There were no complications with the procedure or the anesthetic technique, and the patient was discharged after 10 days.

## Discussion

Our case report delineates the successful use of a bilateral ESP block with catheter placement for postoperative pain management in a partial hepatectomy. To the best of our knowledge, this is the first case report on a continuous ESP block performed with lidocaine for abdominal surgery. The patient indicated satisfactory pain control and no complications. Even though we utilized lidocaine in our study, our findings align with previous reports concerning the effectiveness and safety of ESP blocks in abdominal surgeries such as liver resection [2].

The ESP block represents a relatively novel regional anesthesia technique that entails the injection of local anesthetic into the fascial plane between the erector spinae muscle and the transverse process of the vertebra [1]. This block can provide analgesia to the dermatomes and myotomes supplied by the spinal nerves that traverse the plane of injection [3]. The ESP block carries several benefits, including its simplicity, safety, and adaptability. It can be performed under ultrasound guidance, which can enhance accuracy and diminish complications, and it can be combined with other analgesic modalities for pain management.

The ESP block has been demonstrated to offer effective analgesia in abdominal surgical procedures, such as laparoscopic cholecystectomy, appendectomy, and hysterectomy [4]. Also, studies have attested to its effectiveness in alleviating postoperative pain and reducing opioid usage in patients undergoing liver resection [2].

However, employing the ESP block in liver surgery is not devoid of challenges. A major limitation is the potential for insufficient dispersion of the local anesthetic in certain cases, leading to incomplete analgesia. The ideal volume and concentration of the local anesthetic for the ESP block remain under debate, with the choice possibly contingent on multiple factors, including the patient's age, weight, and comorbidities [3]. Another restriction lies in the need for studies to ascertain the safety and efficacy of catheter placement for continuous infusion of local anesthetic, even though it has been deemed safe for anticoagulated patients. This factored into our decision to choose this approach in our case, given the patient's coagulopathy risk.

In our case, 1% lidocaine was employed due to a lack of long-lasting local anesthetics in the hospital at the time. Despite this limitation, the patient reported a considerable enhancement in pain management compared to his previous surgery, where regional anesthesia was not utilized. These findings suggest that this method may be a feasible alternative for similar circumstances, delivering effective analgesia during both the intraoperative and immediate postoperative periods.

De Cassai et al. conducted a case series illustrating the pharmacokinetics of lidocaine in the context of ESP block for spinal surgery [5]. The study's findings suggest a prolonged elimination half-life for lidocaine in older and obese patients, potentially contributing to a more sustained analgesic effect.

Pain scores at rest remained low throughout the first 24 hours, and opioid consumption was minimal. The absence of PONV is also significant, given that this is a frequent complication associated with opioid usage.

Recent literature has demonstrated that the multimodal approach in contemporary anesthesia can both contribute to intraoperative hemodynamic stability and prevent the development of chronic pain in the postoperative period through the use of regional anesthesia and intravenous adjuvants.

## Conclusions

In this case report, we present a patient who underwent a partial hepatectomy and received a bilateral ESP block with catheter placement for perioperative analgesia using 1% lidocaine. The results demonstrated excellent analgesia and high patient satisfaction. In the context of liver surgery, where neuraxial approaches may be contraindicated due to the risk of coagulation disorders, fascial plane blocks have gained popularity, as they can be employed regardless of this factor. Due to resource limitations, lidocaine was chosen as the local anesthetic, and our findings indicate that lidocaine was safely utilized, providing effective analgesia in this particular case. Comprehensive investigations are needed to thoroughly evaluate the potential advantages of this technique across varied patient demographics and surgical environments.
